# Recombination Is a Major Driving Force of Genetic Diversity in the Anaplasmataceae *Ehrlichia ruminantium*

**DOI:** 10.3389/fcimb.2016.00111

**Published:** 2016-09-29

**Authors:** Nídia Cangi, Jonathan L. Gordon, Laure Bournez, Valérie Pinarello, Rosalie Aprelon, Karine Huber, Thierry Lefrançois, Luís Neves, Damien F. Meyer, Nathalie Vachiéry

**Affiliations:** ^1^CIRAD, UMR CMAEEPetit-Bourg, Guadeloupe, France; ^2^INRA, UMR1309 CMAEEMontpellier, France; ^3^Centro de Biotecnologia-UEM, Eduardo Mondlane UniversityMaputo, Mozambique; ^4^Université des AntillesPointe-à-Pitre, Guadeloupe, France; ^5^Department of Veterinary Tropical Diseases, Faculty of Veterinary Science, University of PretoriaOnderstepoort, South Africa

**Keywords:** *Ehrlichia ruminantium*, MLST, recombination, genetic diversity, genetic population structure

## Abstract

The disease, Heartwater, caused by the *Anaplasmataceae E. ruminantium*, represents a major problem for tropical livestock and wild ruminants. Up to now, no effective vaccine has been available due to a limited cross protection of vaccinal strains on field strains and a high genetic diversity of *Ehrlichia ruminantium* within geographical locations. To address this issue, we inferred the genetic diversity and population structure of 194 *E. ruminantium* isolates circulating worldwide using Multilocus Sequence Typing based on *lipA, lipB, secY, sodB*, and *sucA genes*. Phylogenetic trees and networks were generated using BEAST and SplitsTree, respectively, and recombination between the different genetic groups was tested using the PHI test for recombination. Our study reveals the repeated occurrence of recombination between *E. ruminantium* strains, suggesting that it may occur frequently in the genome and has likely played an important role in the maintenance of genetic diversity and the evolution of *E. ruminantium*. Despite the unclear phylogeny and phylogeography, *E. ruminantium* isolates are clustered into two main groups: Group 1 (West Africa) and a Group 2 (worldwide) which is represented by West, East, and Southern Africa, Indian Ocean, and Caribbean strains. Some sequence types are common between West Africa and Caribbean and between Southern Africa and Indian Ocean strains. These common sequence types highlight two main introduction events due to the movement of cattle: from West Africa to Caribbean and from Southern Africa to the Indian Ocean islands. Due to the long branch lengths between Group 1 and Group 2, and the propensity for recombination between these groups, it seems that the West African clusters of Subgroup 2 arrived there more recently than the original divergence of the two groups, possibly with the original waves of domesticated ruminants that spread across the African continent several thousand years ago.

## Introduction

*Ehrlichia ruminantium* is an intracellular bacterium responsible for heartwater, an important and fatal tropical disease of wild and domestic ruminants (Allsopp, 2009; Moumene and Meyer, 2016). This bacterium is transmitted by *Amblyomma hebraeum* ticks in southern Africa and by *Amblyomma variegatum* ticks, the most wide spread vector through sub-Saharan Africa, Indian Ocean islands and the Caribbean (Walker and Olwage, 1987).

Heartwater occurs in almost the whole of sub-Saharan Africa (except for the very dry southwest), in São Tomé and Principe and in the Indian Ocean islands of Madagascar, Zanzibar, Mayotte, Mauritius, Reunion islands, and the Comoros (Provost and Bezuidenhout, 1987; Stachurski et al., 2013). The disease is also present in the three Caribbean islands of Guadeloupe, Marie Galante, and Antigua and it represents a threat to the American mainland (Barré et al., 1987; Roth et al., 2013).

As of yet, no efficient single vaccine against heartwater is available due to a limited cross protection between vaccinal and field strains, which is probably caused by the high genetic diversity of *E. ruminantium* in any given geographical location (Vachiéry et al., 2013).

In order to better define appropriate control strategies against heartwater, it is important to understand the diversity of *E. ruminantium* strains within regions, their evolution and origin of introduction as well as to attempt to associate *E. ruminantium* genotypes with possible protective genetic markers. Presently, the genetic diversity of *E. ruminantium* has been elucidated through polymorphic and conserved genes such as *map1, 16S* rRNA, and some housekeeping genes for a limited number of strains.

Allsopp and Allsopp (2007) studied the genetic diversity of 12 different *E. ruminantium* strains from Africa and the Caribbean using a panel of core function and housekeeping genes: *16S rRNA, gltA, groEL, ftsZ, sodB, nuoB, rnc* (*pCS20*), and *ctaG* (*pCS20*). This study highlighted inconsistent phylogenies for the different genes examined and evidence for recombination and a separation of strains into two clades, South-East Africa, and West Africa (Allsopp and Allsopp, 2007).

Using *map1* and *map1* family genes to genotype *E. ruminantium* strains, several authors showed a high genetic diversity at local (Gambia and Burkina Faso), regional and worldwide scales (Caribbean region, Africa, and Madagascar; Faburay et al., 2008; Vachiéry et al., 2008; Adakal et al., 2010b; Raliniaina et al., 2010). In localized areas of Gambia and Burkina Faso, the studies observed at least 11 sequence types and mixed infections with several *E. ruminantium* strains in ruminants and ticks. In the Caribbean, another study using the *map1* gene demonstrated a high diversity of *E. ruminantium* strains with nine different genotypes present either at the scale of locality, islands and region (Vachiéry et al., 2008). Furthermore, comparison of strains isolated in Africa, in the Caribbean and in Madagascar using the *map1* gene family, revealed a divergent evolution for this gene in *E. ruminantium* (Raliniaina et al., 2010). Divergent evolution was shown by the identification of different genotypes for each *E. ruminantium* strain depending on the *map1* paralogs used. Furthermore, important *map1* diversity was conserved independently of sampling scale (village, region, continent) and the timing of introduction (punctual for Caribbean strains vs. continuous introduction for African strains).

In general, *map1* gene appeared to be a good tool to characterize the genetic diversity among Africa, Caribbean and Madagascar, however, there was a lack of correlation between *map1* genotype and geographic origin (Raliniaina et al., 2010) and limited cross protection between strains (Adakal et al., 2010b).

In order to construct *E. ruminantium* phylogeny and elucidate genetic population structure, a multilocus sequence typing (MLST) analysis based on housekeeping genes (Adakal et al., 2009, 2010a; Nakao et al., 2011) and a multilocus variable number of tandem repeats based on mini-satellites were developed for *E. ruminantium* (Pilet et al., 2012). The apparent advantages of these techniques are based on a perceived lack of ambiguity, portability, reproducibility, good discriminatory powers to differentiate isolates and ability to be automated (Lindstedt, 2005; Sullivan et al., 2005). MLST can overcome certain challenges to phylogenetic and phylogeographic reconstruction and potentially allows the study of the diversity of the bacterium, which could aid in the design of efficient vaccines that includes appropriate local and/or regional strains (Urwin and Maiden, 2003; Lindstedt, 2005; Sullivan et al., 2005). However, inference of bacterial evolution and population genetics can be complicated by the presence of recombination.

Recombination is an exchange of genetic material between organisms or chromosomes to form new combinations of genetic material on a chromosome (Smith et al., 1993; Martin and Beiko, 2010). The unaccounted for presence of recombination can lead to an overestimation of population expansion, the false detection of positive selection (Schierup and Hein, 2000; Shriner et al., 2003) and to the reconstruction of an erroneous phylogeny (Posada and Crandall, 2002; Ruths and Nakhleh, 2005). Recombination can be detected by several means, including a lack of congruence in phylogenetic trees (Feil et al., 1996; Zhou et al., 1997), mosaic-like DNA sequences (Spratt et al., 1995), excess of homoplasy in phylogenetic trees (Smith and Smith, 1998) and a network relationship between sequences, using split decomposition (Holmes et al., 1999).

Recombination occurrence has also been described for *E. ruminantium* in studies done by Allsopp and Allsopp (2007), Bekker et al. (2005), Hughes and French (2007), and Nakao et al. (2011). Up to now, the implications of recombination for *E. ruminantium* as a major driver of its genetic diversity have been given little attention. Moreover, high genetic diversity is probably tightly associated with limited cross-protection between vaccinal and some field strains (Jongejan et al., 1991; Allsopp and Allsopp, 2007; Vachiéry et al., 2013). This omission could be important in the interpretation of the results of previous studies attempting to reconstruct the relationship and population structure between *E. ruminantium* strains.

In order to understand the genetic diversity and population structure of 194 worldwide *E. ruminantium* isolates, MLST analysis using *lipA, sucA, sodB, secY*, and *lipB* core function and housekeeping genes was performed. The presence of recombination with the presence of reticulations in a split-decomposition network and tests of recombination within and between different subgroups in the network using the pairwise homoplasy index (PHI) test as well as incongruence in gene trees for these five genes was demonstrated. These results show the relevance of recombination events in the generation of *E. ruminantium* diversity and evolution. Two major genetic groups, a West African cluster and a worldwide cluster which includes West Africa, East Africa, Southern Africa, Indian Ocean, and Caribbean, could be delineated by MLST. The prevalent recombination events observed in the current study suggest a complex population structure for *E. ruminantium* strains and suggests that caution should be taken when reconstructing relationships in closely related species in the *Anaplasmataceae*, especially if using only a subset of the genome, like in MLST analyses.

## Materials and methods

### Field collection of ticks and blood samples

*A. hebraeum* and *A. variegatum* ticks were collected from cattle during epidemiological studies in the south and center of Mozambique from 2011 to 2013 and Indian Ocean Islands (Reunion Island, Comoros Islands, Mayotte, and Madagascar) from 2007 to 2010 and chosen randomly for each animal sampled. Blood samples of heartwater clinical cases from Guadeloupe were provided to CIRAD by the surveillance network monitoring ruminant neurological syndromes (RESPANG) with the collaboration of the veterinary services. Samples from RESPANG were collected following an ethical committee approval from 2011 to 2014. Ticks were stored in 70% ethanol at room temperature and taxonomically identified as being *A. variegatum* and *A. hebraeum*. Blood was preserved either frozen at −20°C or in 70% ethanol at room temperature until DNA extraction. All samples were tested for *E. ruminantium* positivity using *pCS20* nested PCR as described below.

### *pCS20* nested PCR and multilocus sequence typing

DNA was extracted from tick tissues and blood using the QiaAmp DNA minikit (Qiagen, Courtaboeuf, France) in accordance with manufacturer's instructions. Detection of *E. ruminantium* was performed using a semi-nested PCR for a fragment of the *pCS20* gene, as previously described by Molia et al. (2008). For the *pCS20* nested PCR, a first PCR phase was performed using the primer pair AB128′/AB130′ and for the second phase the primer pair used was AB129′/AB130′. Amplified products were visualized on a 1.5% agarose gel (TAE buffer) and considered positive if exhibiting a band of 280 bp. Positive samples were then typed using a modified Multilocus sequence typing scheme which includes a panel of five genes instead of eight. Five variable housekeeping genes, *lipA, lipB, secY, sodB*, and *sucA* were amplified using the primers and PCR conditions previously described by (Adakal et al., 2009, 2010a). PCR products were sequenced by Beckman Coulter Genomics (France).

### *E. ruminantium* isolates

A total of 194 isolates originating from several geographical areas were analyzed by MLST: North and Central Africa (2), West Africa (55), Eastern Africa (3), Southern Africa (64—mainly from Mozambique), Indian Ocean Islands (29), and Caribbean (41—mainly from Guadeloupe; Tables S1, S2). The geographical origin of strains and isolates, their number and name, reference and date of isolation are shown in Table S2. Previously published DNA sequences from Burkina Faso, Caribbean, and Madagascar extracted from tick tissue, blood and cell culture as well as some reference strains were also used (Adakal et al., 2010a; Raliniaina et al., 2010; Nakao et al., 2011). The number of isolates per country is shown in Table S1. The main sampling areas were Mozambique (58 isolates, 30% of sampling) for Southern Africa, Burkina Faso (44 isolates, 23% of sampling) for Western Africa, Guadeloupe (40 isolates, 21% of sampling) for the Caribbean and Madagascar (13 isolates, 7% of sampling) for the Indian Ocean.

### Data analysis

All the Figures were edited using Inkscape software (Harrington, 2004–2005). Multiple sequences were aligned and edited to produce a consensus and concatenated sequence for each strain using the software Geneious 8.1.7. R8 (Kearse et al., 2012) and AliView 1.17.1 (Larsson, 2014). Sequences were concatenated in the order *sucA*-*sodB*-*lipA*-*secY*-*lipB* resulting in a final sequence of 2314 bp length. If a set of sequences were identical for some isolates (clones), only one representative sample of each group was included in the bioinformatics analysis. Bayesian trees were generated with BEAST (Drummond et al., 2012). The dataset was partitioned into one partition per gene alignment and the trees were linked over the five partitions. The site model was set to the “BEAST Model Test,” testing all reversible models and estimating the mutation rate (initial value was set to 0.001). A strict clock and an exponential population growth coalescent model were used. The Markov Chain Monte Carlo was run for 10,000,000 generations on two occasions to ensure correct mixing, and the resulting log files was reviewed in Tracer (Rambaut et al., 2014). The 95% Highest Posterior Density (HPD) for the growth rate did not contain 0, allowing the rejection of constant population growth. The Effective Sample Size (ESS) for four of the five tree likelihoods was less than 100, and examination of the traces revealed that they did not converge properly. For visualization of the trees with Densitree (Bouckaert, 2010), 25% of samples were removed from burn-in. The resulting image of 7.5 million overlaid trees and the “root canal” tree which represents the total set is shown in **Figure 2**.

The alignment of concatenated sequences from the five genes (*sucA, sodB, lipA, secY*, and *lipB*) was imported to SplitsTree4 program version 4.13 and a phylogenetic network was constructed using the neighbor-net algorithm (Huson and Bryant, 2006).

Maximum likelihood phylogenies were constructed using a concatenated alignment of the five genes with PhyML (Guindon et al., 2010) with and without *E. chaffeensis* as an outgroup under the GTR+G+I model of evolution with four rate categories and aLRT (approximate Likelihood-Ratio Test)-based branch support.

Determination of genetic groups in the network was done by combined inspection of the multiple sequence alignment, split decomposition network and a heat map, which represents the degree of relatedness between strains (genetic distance). Heat maps allow the visualization of similarity and differences in the data by representing values contained in the distant matrix as colors in a graph. The heatmap was used to identify possible recombinants by finding sequence types with atypical similarity to others from outside their group. The heat map was generated using the heatmap tool, “heatmap.2” of the “gplots” (Warnes et al., 2009) package of the R software based on a distance matrix calculated using the Tamura and Nei (1993) model from the “ape” (Paradis et al., 2004) package (R Core Team, 2013). Dendograms representing hierarchical clusters of sequence types and their sequence type numbers are shown along the axes of the heatmap.

Additionally a population structure analysis was performed using STRUCTURE (Pritchard et al., 2000). The analysis was performed with an admixture model with correlated genotypes for *K* = 2 to *K* = 9, with burnin period of 10,000 MCMC generations followed by 100,000 MCMC generations. For each K, 50 iterations were performed, and the optimal K was calculated using the Evanno method (Evanno et al., 2005) on the STRUCTURE Harvester webserver (Dent and von Holdt, 2012). CLUMP (Jakobsson and Rosenberg, 2007) was used to find the optimal clustering from the multiple iterations.

The (Pairwise Homoplasy Index) PHI test (Bruen et al., 2006) was conducted to determine whether recombination events were present in the concatenated sequence alignment. A PHI test was also performed within and between determined groups and *p*-values for each comparison are presented in Table 1. The *p*-values were corrected for multiple testing using Benjamini-Hochberg FDR method (Benjamini and Hochberg, 1995; available online at: http://www.sdmproject.com/utilities/?show=FDR). Recombination within and between genetic groups was considered positive if the PHI test yielded a corrected *p* ≤ 0.05.

**Table 1:** **Phi test for recombination from two genetic groups (1 and 2) and five subgroups (2A, 2B, 2C, 2D, and 2E) of *E. ruminantium***.

**Genetic group**	**Corrected *p*-values**
1	1.00
2	0.00^*^
2A	N/A
2B	1.00
2C	0.83
2D	0.03^*^
2E	0.06

N/A, not applicable because all the genotypes are recombinants;

* *Positive for recombination, PHI test yielded a p ≤ 0.05*.

ClonalFrame (Didelot and Falush, 2007) was used to estimate the ratio of recombination to mutation (r/m).

## Results

### *E. ruminantium* population genetic structure

DNA sequences were deposited in GeneBank and accession numbers are displayed in Table S4.

**Figure 1:**
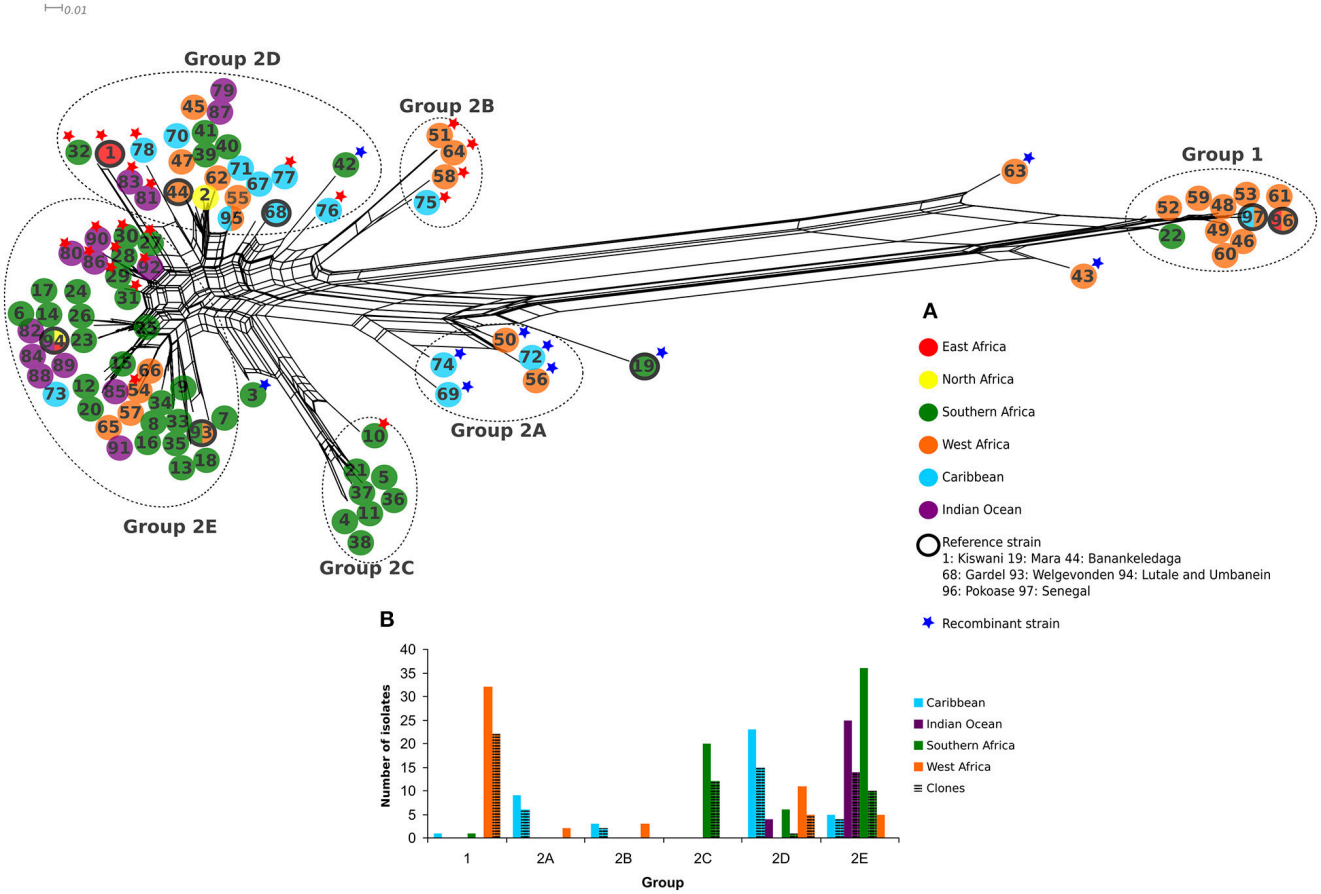
**(A)** Split decomposition network of 97 *E. ruminantium* genotypes obtained from five concatenated housekeeping genes. Strains are color coded according to the geographic origin described in the legend. Reference strains are identified by black circles. Recombinant strains are tagged with a blue star, and those with less than 80% inferred ancestry from any single population in STRUCTURE with *K* = 5 are tagged with a red star. All recombinants also had less than 80% ancestry from any given population. Two main genetic groups and five subgroups are described as Group 1, 2A, 2B, 2C, 2D, and 2E. **(B)** Histogram representing the total number of *E. ruminantium* isolates sampled per geographical origin for each genetic group.

**Figure 2:**
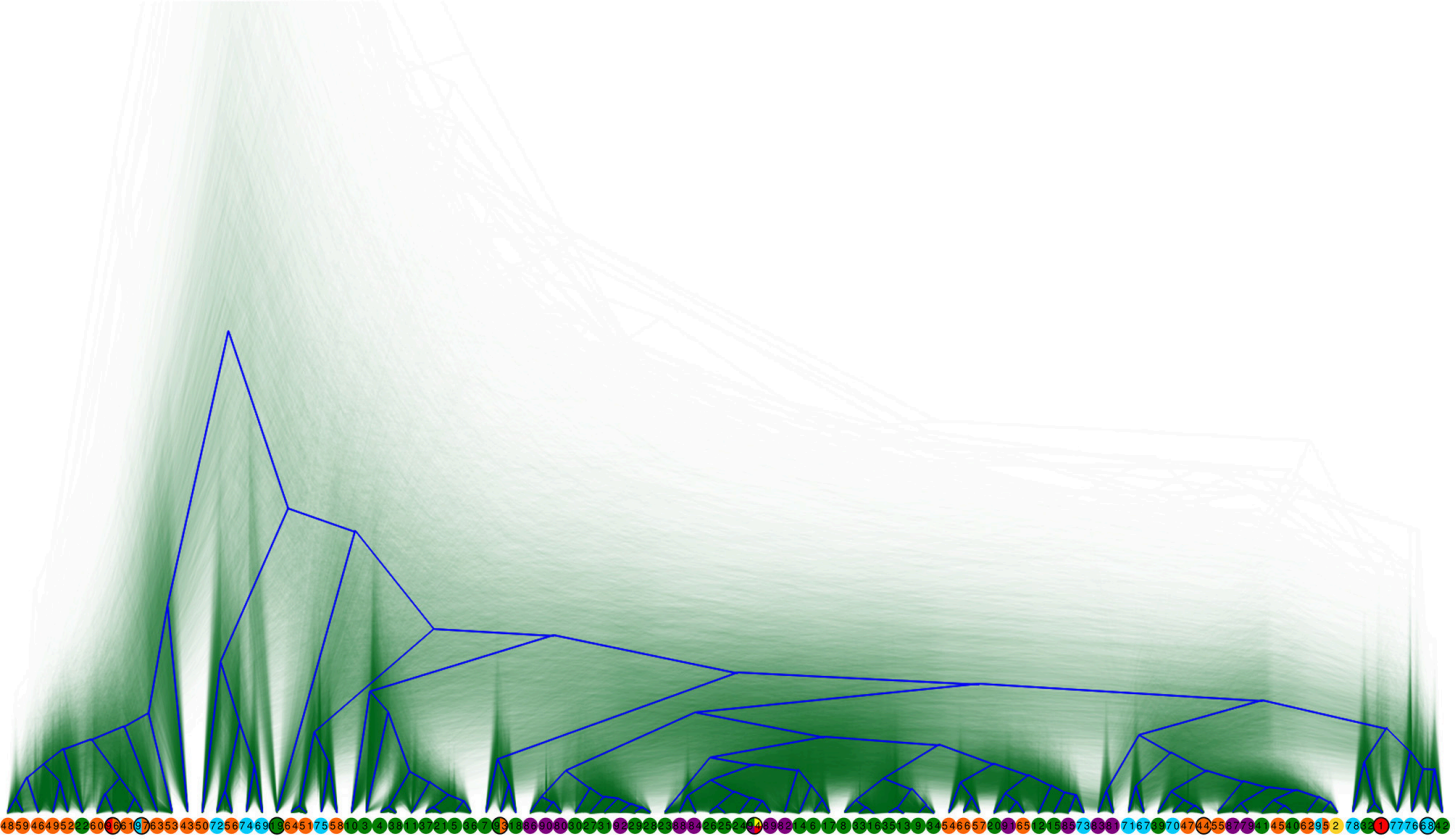
**Overlaid Bayesian consensus trees of 97 *E. ruminantium* genotypes generated using BEAST and visualized in Densitree**. The root canal tree is shown in blue. Sequence types at tips are color coded according to the geographic origin: East Africa (red), North Africa (yellow), Southern Africa (green), West Africa (orange), Caribbean (blue), Indian Ocean (purple). Reference strains are identified by black circle outlines.

Concatenated sequences are based on five housekeeping genes. From 194 *E. ruminantium* isolates, only 97 representing unique sequence types are shown in the phylogenetic network and tree (Figures 1A, 2, Figure S1). The remaining 97 *E. ruminantium* isolates were clonal to these sequence types for the genes examined. Several isolates, representing one sequence type from the same geographical origin, are shown by a circle with a single color, whereas clonal isolates from different geographical origins were divided into several colors within one circle (Figures 1A, 2).

**Figure 3:**
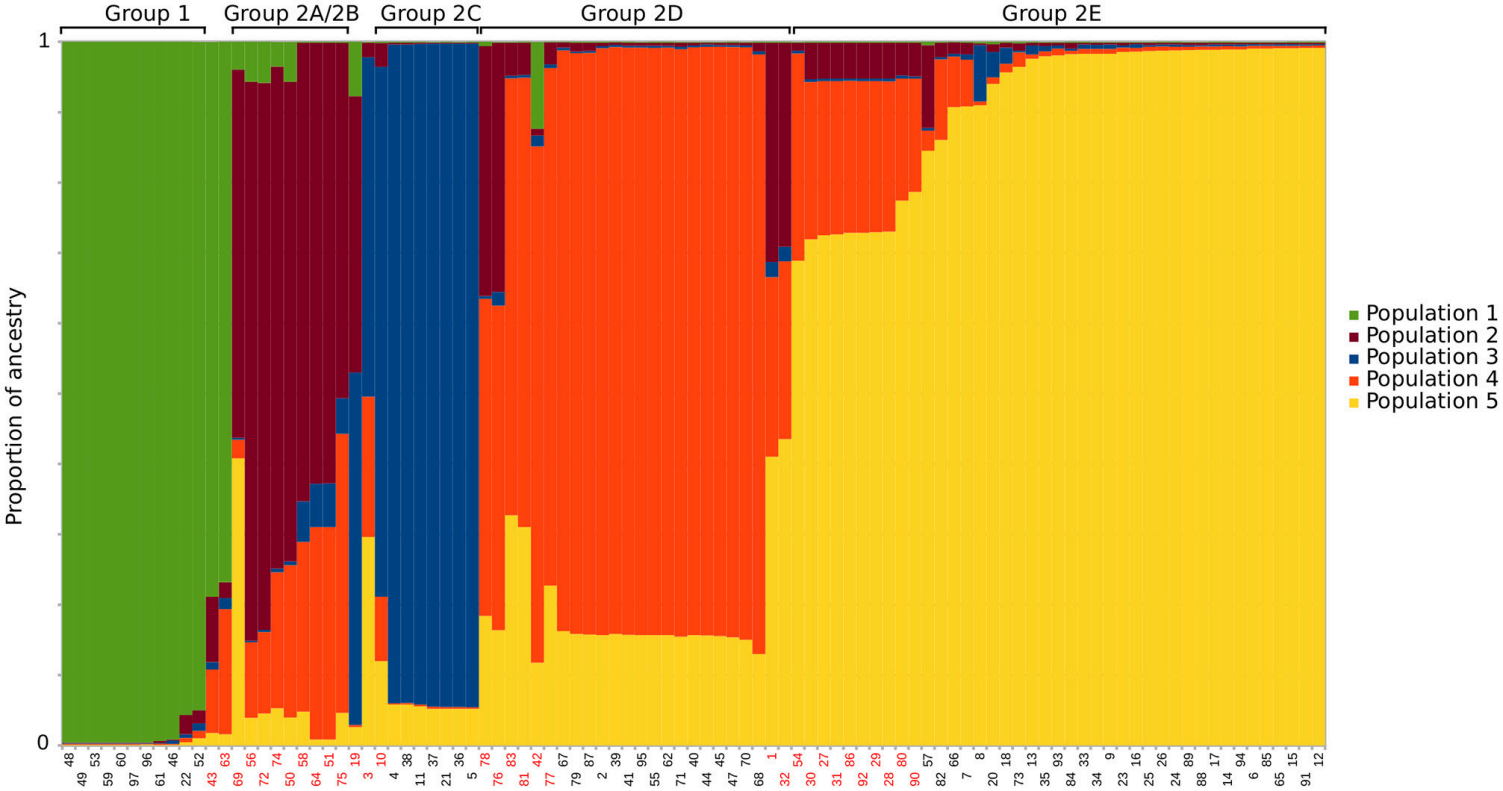
**STRUCTURE analysis for *K* = 5 using an admixture model and correlated genotypes**. Each column represents the inferred ancestry for each sequence type from the five predicted populations. The groups from the hierarchical clustering and splitstree analysis are noted above the columns. Sequence types with less than 80% inferred ancestry from a single population have red labels.

A split decomposition network was constructed from 97 concatenated *E. ruminantium* sequences and illustrated in Figure 1A. The split decomposition network resembled a network-like structure composed of two distinct and divergent groups: Group 1 (West Africa) and a Group 2 (worldwide) with West/East/Southern Africa, Indian Ocean, and Caribbean strains (Figure 1A). Reticulations in the graph are clearly present (especially noticeable in Group 2), and are evidence for recombination or other forms of homoplasy between different sequence types. Group 2 is composed of five subgroups, which were defined based on arrangement of sequence types in the network (Figure 1A), a hierarchical clustering (heat map: Figure S2), and careful examination of the multiple sequence alignment (Figure S3). Several sequence types were excluded from groups due to being recombinants identified by examination of the heat map and multiple sequence alignment (marked with blue stars in Figure 1A, Figure S3). In the STRUCTURE analysis (Figure 3) the optimal *K*-value was found to be 2, which is not unexpected given the long branch separating Group 1 and Group 2. When discounting *K* = 2, the next identified optimal grouping is *K* = 5, which is one less group than we defined using the other methods. The vast majority of sequence types are placed in equivalent groups between the two analyses, however the STRUCTURE analysis combines subgroup 2A and subgroup 2B, and clusters a few other strains in with those from different groups. In these cases, the strains tend to have a large proportion of inferred admixture (in this case equating to possible recombination), for example, ST 32 and ST 1 appear to show slightly more ancestry with the group equating to subgroup 2E instead of 2D, ST 69 from subgroup 2A clusters with subgroup 2E sequences, however both have evidence of ancestry from both subgroups.

The histogram in Figure 1B represents the total number of *E. ruminantium* isolates including unique sequence types and clones and only clones, per geographical origin belonging to Group 1 and Group 2. Group 1 was represented mostly by West African strains (32 isolates; Figures 1A,B). Twenty-five isolates were from Burkina Faso, three from Senegal, two from Ghana and one isolate from Gambia, Guadeloupe, Nigeria, Tanzania, and Mozambique (Table S3). Sequence type 97 included 17 isolates from West Africa (with the reference strain Senegal) and one from the Caribbean whereas sequence type 96 included one isolate from East Africa and the reference strain Pokoase from West Africa.

**Figure 4:**
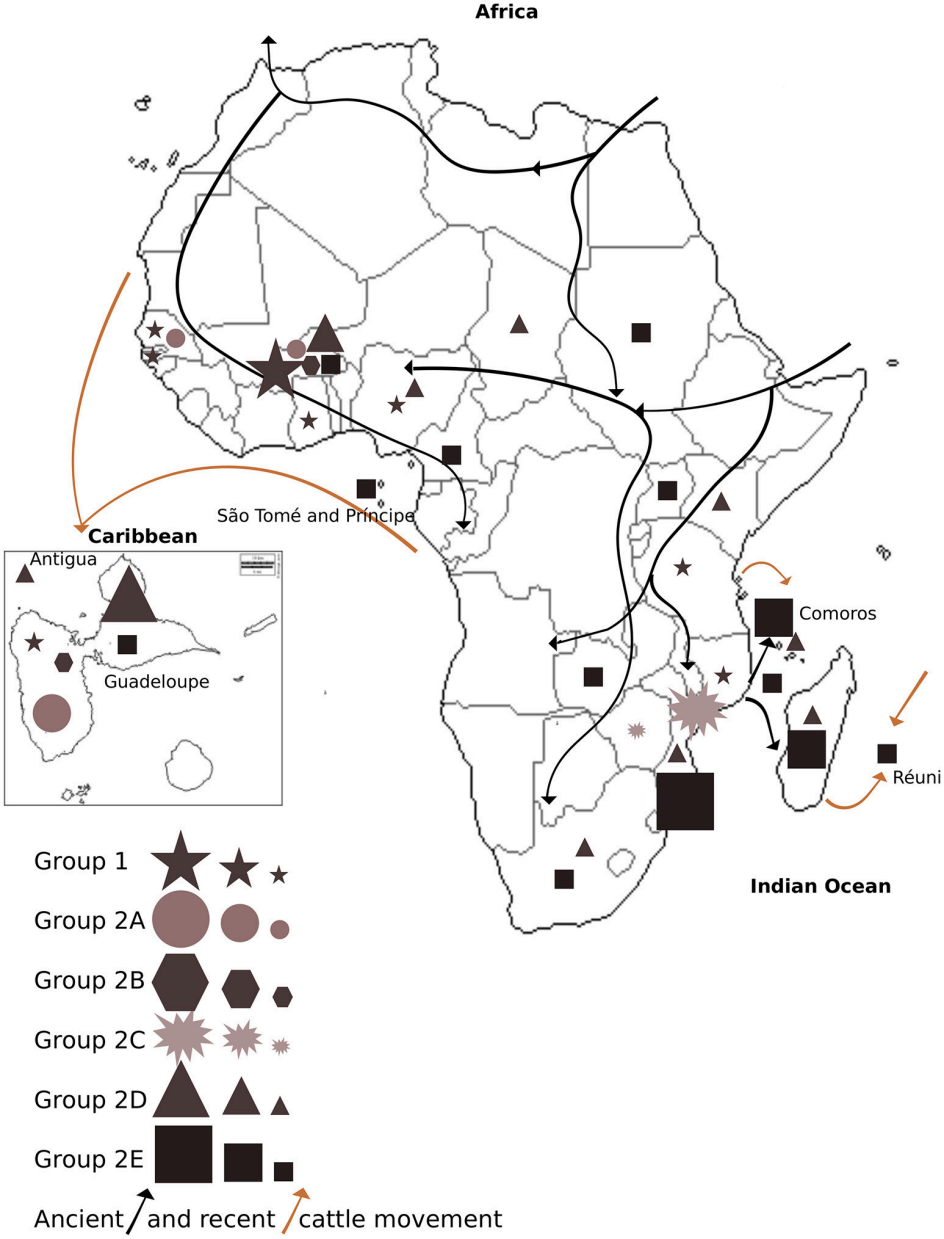
**Distribution of *E. ruminantium* genetic groups and subgroups 1, 2A, 2B, 2C, 2D, and 2E in each sampled country within Africa, Caribbean, and Indian Ocean Islands**. Groups are coded by symbols according to the legend. Symbol size corresponds to sampling size defined by the following sample threshold: >15 samples (big symbol), < 15 samples (medium symbol), and < 5 samples (small symbol). Recent (< 400 years ago; brown arrows) and ancient (>400 years ago; black arrows) movement of cattle is represented in the map.

The Group 2 (worldwide) was diverse and composed of 82 unique sequence types from all the sampled geographical areas (Figure 1A). All of these subgroups with the exception of subgroup 2D have more frequent representation of certain geographical regions in our data. Subgroups 2A and 2B were composed of isolates from West Africa and Caribbean. Group 2A is present in Burkina Faso, Senegal, and Guadeloupe, with Guadeloupe containing most of the isolates (Figures 1B, 4, Table S3). Group 2B is represented in Burkina Faso and Guadeloupe, with equal number of isolates for each group (Figures 1B, 4, Table S3). Subgroup 2C was exclusively composed of Southern African isolates (20 strains), the majority being from Mozambique and one, Crystal Spring strain, from Zimbabwe (Figures 1B, 4, Table S3). Subgroups 2D is the most geographically diverse subgroup at a regional scale with representatives from each large scale region considered. The majority of the strains in this subgroup are from the Caribbean (23 strains) and West Africa (11 strains; Figure 1B). Subgroup 2D is represented in 10 countries (Antigua, Burkina Faso, Chad, Comoros, Guadeloupe, Kenya, Madagascar, Mozambique, Nigeria, South Africa; Figure 4, Table S3). Sequence type 95 (subgroup 2D) includes two strains from West Africa and four from the Caribbean (Figure 1, Table S2). The reference strains Kiswani (Kenya), Banankeledaga (Burkina Faso), and Gardel (Guadeloupe) are part of subgroup 2D. Upon close examination of the alignment, some of the sequence types from Southern Africa (39, 40, and 41) and the Indian Ocean (79 and 87) are differentiated simply by gaps caused by lack of coverage at the end of certain gene sequences or by one residue unique to a sequence, likely increasing the representation of their regions in the group in terms of unique sequence types (Figure S3). Strains from West Africa and the Caribbean tend to be more genetically diverse within this group. Notable exceptions to the lack of diversity in Southern African sequence types in this subgroup are sequence type 42, which is recombinant as noted below, and sequence type 32, which is nearly identical to the East African sequence type 1 (Kenya), and shares its *lip*B gene with West African and Caribbean subgroup 2B types 51 and 64. They also share a *sucA* allele (that is otherwise exclusively found in subgroup 2D) with two Indian Ocean sequence types 83 and 81 in this subgroup.

Subgroup 2E is dominated by Southern African (36 strains), mainly Mozambique, and Indian Ocean isolates (25 strains) with a small number of other sequence types from West African or mixed origin. This subgroup is present in 13 countries (Burkina Faso, Cameroon, Comoros, Guadeloupe, Madagascar, Mayotte, Mozambique, Reunion, São Tome e Principe, South Africa, Sudan, Uganda, Zambia; (Figures 1B, 4, Table S3). Sequence type 93 (subgroup 2E) had one isolate from West Africa and seven from Southern Africa (Figure 1, Table S2). Sequence type 94 (subgroup 2E) contains one isolate each from North Africa, East Africa and Southern Africa and two isolates from the Indian Ocean. The reference strains Welgevonden (South Africa), Lutale (Zambia) and Umbaneim (Sudan) are part of the subgroup 2E (Figure 1, Table S2).

Two sequence types from Burkina Faso (43, 63) and two sequence types from South Africa (3, 19 corresponding to reference strain Mara) were not part of any genetic group as they are recombinant strains (Figure 1A). Recombination was identified based on the split decomposition network location (usually at the vertices), and examination of the multiple sequences alignment and the heat map for close similarity between the given sequence type and a different subgroup (Figure S2). Several other sequence types (50, 56, 69, 72, and 74) from subgroup 2A and sequence type 42 from subgroup 2D tagged with a blue star in Figure 1A were also identified as recombinants based on combined inspection of the multiple sequence alignment, heat map and split decomposition network (Figure 1A, Figures S2, S3).

The distribution of *E. ruminantium* genetic groups in the sampled countries as well as the recent (less than 400 years ago) and ancient (more than 400 years ago) cattle movement (domestication) in Africa, the Caribbean an Indian Ocean Islands are shown in Figure 4, Table S3.

### Evidence of recombination events for *E. ruminantium*

A Bayesian MCMC phylogeny created with BEAST using the five gene partitions with the 97 nucleotide sequences in each partition highlighted conflicting topologies (Figure 2). Reviewing the trace files revealed that the ESS of the four linked trees were all less than 100 and the trace plots reveal that they did not converge properly. This indicated conflicting signals in our data, which are also illustrated by low branch support values in the maximum likelihood trees (Figure S1). In phylogenetics, low branch support can be a quantitative indication of recombination or other forms of homoplasy in the data and is also common for short branches. In this case the conflicting signals are most likely due to recombination events between the analyzed strains given the evidence of mosaic genes in the alignment, the reticulate structure in the split decomposition network (Figure 1) and the mixed ancestry exhibited in the STRUCTURE analysis (Figure 3). The overlaying of all the trees produced by the BEAST analysis after discarding for burn-in reveals fuzzy regions in the tree which indicate conflicts in the reconstruction. *E. ruminantium* sequence types are widespread and the tree did not show a clear phylogeographic structure between Africa, Indian Ocean islands and the Caribbean, although several isolates from the same or close geographical origin cluster together as described above (Figures 1, 2). The ratio of recombination to mutation was estimated as 1.351 ± 0.0167, (any given residue difference between two sequences, is 1.351 times more likely to have been introduced by recombination than by mutation). The PHI test on 194 *E. ruminantium* isolates found statistically significant evidence for recombination (*p* = 0.0). Furthermore, recombination analysis between the six genetic groups and subgroups was mostly positive and only intragroup recombination was represented in Table 1. Examination of the alignment reveals clear mosaicism (blocks of multiple residues shared with a different group to the exclusion of other members of the same group) in sequence types 63, 43, 19, 42, 3 and all strains in subgroup 2A (Figure S3). These are the most obvious events because they are between regions that are divergent that contain several mutations delineating the recombination breakpoints. The strains in general do not contain many variable sites in the genes used, and there are many possible cases where recombination might transfer single SNPs. In such cases it is difficult to identify the events with certainty because the possibility of homoplasy by convergent mutation exists. The PHI test does not identify recombination breakpoints, but does allow a statistical evaluation of the likelihood of recombination between different sequences regardless of the size of the recombined blocks. This type of test is advantageous in cases where the sequence similarity is high and there are only a small number of discriminatory SNPs to infer recombination compared to methods that attempt to identify breakpoints at the edges of recombination blocks because it may detect the overall signal of recombination from a series of small events that are undetectable individually.

The STRUCTURE analysis (Figure 3) also hints at recombination in the groups, signified by the presence of many sequence types that are inferred to have ancestry from more than one different population in the *K* = 5 analysis. In all, 50 of the 97 sequence types have inferred ancestry of less than 90% from any one population, while 32 have less than 80% and five sequence types have less than 50% inferred ancestry from any single population. We have marked sequence types that have less than 80% inferred ancestry from a single population with red stars in Figure 1A, Figure S3. Although STRUCTURE doesn't provide a statistical measure for the likelihood of recombination between sequence types, the analysis does suggest widespread recombination between the five different inferred populations.

## Discussion

### *E. ruminantium* phylogeny and genetic population structure

Despite the apparent unclear population structure and phylogeography of *E. ruminantium* strains, two main distinct genetic groups were defined: Group 1 (West Africa) and a worldwide Group 2, the latter being represented by West, East and Southern Africa, Indian Ocean and Caribbean isolates. Outgrouping the tree using *E. chaffeensis* (Figure S1A) reveals that the root of the *E. ruminantium* species divides Group 1 from Group 2. The branches leading to each group are relatively long compared to the branches within the groups, allowing us to infer that the groups were probably isolated from one another for a long time. We also infer the possible geographical locations of the ancient progenitors of the groups based on the geographic distributions of the extant strains: we propose that the Group 1 is ancestrally West African, and that Group 2 originates elsewhere. The divergence of the two main groups possibly predates the spread of livestock across the continent of Africa with the wave of domestication. The presence of several individual sequence types and all sequence types present in subgroup 2A that represent recombination between Group 1 and Group 2 in West Africa, shows the propensity of these groups to recombine. This allows us to infer a later arrival of subgroup 2D in West Africa long after the split between the two groups otherwise the clusters would be more homogeneous.

### Influence of wildlife, ticks, and ancient cattle movement on *E. ruminantium* population structure

Apart from *Anaplasma marginale* (de la Fuente et al., 2007; Estrada-Pena et al., 2009), no other studies have yet elucidated the influence of wildlife, cattle, and small ruminant movements on the genetic diversity of the *Anaplasmataceae* family.

We hypothesize that ancient (more than 400 years ago) and recent cattle (less than 400 years ago) movements has shaped *E. ruminantium* diversity by creating different genetic groups and subgroups as well as facilitated recombination between diverse strains. Recent and ancient cattle movement (domestication) in Africa, the Caribbean, and the Indian Ocean islands are illustrated in Figure 4. Group 1 and subgroups 2A, 2B, 2D, and 2E present in West Africa are also present in Guadeloupe and Antigua, reflecting the historical movement of cattle from this region to the Caribbean (Maillard et al., 1993). In addition, Indian Ocean strains from subgroup 2E, reflect the ancient movement of cattle from East and Southern Africa to Islands in the Indian Ocean. Subgroups 2D and 2E in Africa cover most of the sampled countries and might reflect the ancient movement of cattle from North and Central-East to West Africa and from East Africa to Southern Africa. Subgroup 2E is predominantly present in Southern Africa and the Indian Ocean, suggesting that the small number of strain types from this group that are present in other locations may be the result of recent cattle movement.

Given that all the current known vectors of *E. ruminantium* are African *Amblyomma* species (Walker and Olwage, 1987) and that these species have as primary hosts large African wild mammals (Voltzit and Keirans, 2003), it is reasonable to infer that well adapted *E. ruminantium* strains were circulating in Africa for thousands of years, before the introduction of the first domestic ruminants. Several studies have reported a South and East genetic break in African mammals (Pitra et al., 2002; Lorenzen et al., 2010) that can possibly help to explain the large genetic distance between Group 1 and Group 2 seen in *E. ruminantium* population. Because of limited sampling in some regions (particularly Central and East Africa) and the lack of a well-established molecular clock for *E. ruminantium* (Hughes and French, 2007), as well as the presence of recombination it is not possible to get information on the time of divergence between *E. ruminantium* strains and genetic groups.

The introduction and expansion of *Bos taurus*, small ruminants and *Bos indicus* in Africa between 8000 and 2500 years ago (Hanotte et al., 2002; Ajmone-Marsan et al., 2010) exposed naïve domesticated ruminant populations for the first time to African *Amblyomma* species and *E. ruminantium* strains. The spill over of *E. ruminantium* strains from African wildlife species to domestic ruminants was arguably associated with high mortality and triggered a long process of host-pathogen adaptation that putatively included intense recombination among *E. ruminantium* strains. We propose that this introduction and spread of domesticated animals are the main trigger for recombination between Group 2 (subgroup 2D) and Group 1.

Archaeological and genetic data on the origin and expansion of domestic ruminant populations in the African continent strongly supports the existence of migratory routes East-West, West-East (less common), North-South and more recently South-North (Hanotte et al., 2002). This domesticated host migratory pattern would support the possibility of pathogen gene flow across the above mentioned migratory routes. In the last millenium (more recently) the development of agriculture and iron technology led to a more sedentary behavior of sub-Saharan Bantu communities with the consequent progressive reduction of trans-continental migrations (Blench and MacDonald, 2000). This fact might have contributed to the gradual isolation of domestic ruminant populations and consequently a reduction in the continental gene flow of vectors and pathogens.

Cattle movement, contact with wildlife coupled with *Amblyomma* tick dispersion, have probably shaped the genetic diversity of *E. ruminantium*. However, the influence of vector species on the strain diversity is unknown. It is worth noting that the genetic diversity of the tick vector *A. variegatum* seems to follow a similar pattern that those of *E. ruminantium*.

For instance, Beati et al. (2012) studied the genetic diversity of *A. variegatum* throughout the Caribbean and Africa and found a West Africa-Caribbean clade and an Eastern Africa clade. Later, Stachurski et al. (2013) found a worldwide clade and an East Africa-Indian Ocean clade by studying the phylogeographic structure of *A. variegatum* in the Indian Ocean. *A. variegatum* genetic clusters seem to generally match *E. ruminantium* genetic groups found in the current and previous studies of Allsopp and Allsopp (2007), Vachiéry et al. (2008), and Nakao et al. (2011). Even if there are similarities between *A. variegatum* and *E. ruminantium* clusters for Caribbean/West Africa isolates and Indian Ocean/Southern Africa and South/Eastern African isolates, the phylogeography of *Amblyomma* ticks appears different from that of *E. ruminantium.*

In the Caribbean and Indian Ocean, it is very likely that *A. variegatum* and *E. ruminantium* were introduced simultaneously with cattle. In the Caribbean, studies on cattle genetic diversity found a correspondence between genetic diversity and historical domestication (importation from West Africa) of cattle from this region (Magee et al., 2002). Also, the genetic diversity of *E. ruminantium* strains (Vachiéry et al., 2008 and our results) linked the sequence types with several origins in Africa, thus suggesting that strains were introduced with *A. variegatum* in the nineteenth century.

### Influence of recent cattle movement on *E. ruminantium* population structure

In cases where a subgroup is dominated by certain geographical regions but is rarely found elsewhere, the parsimonious explanation is that the atypically located strains are the result of recent transport of a strain whose ancestry lies in the region that is predominant in the group, barring sampling biases. As we have large samples from Mozambique, Burkina Faso and Guadeloupe, the origins of subgroup 2D appear puzzling because the frequency of apparently unique sequence types from the four major geographically sampled regions are quite similar.

However, when taking into account the nearly clonal (39, 40, 41 and 79, 87) and recombinant (42 and 32) sequence types from Southern Africa and the Indian Ocean, the majority of the genetic diversity in this subgroup is found in West Africa and the Caribbean. Because livestock transport was likely unidirectional to the Caribbean from West Africa (Hanotte et al., 2002), we can consider that subgroup 2D is thus probably ancestrally West African and the presence of Southern African and Indian Ocean strains within this group may be due to recent movement of the strain types from West Africa to those regions.

The predominance of unique sequence types in subgroup 2D from West Africa/Caribbean suggests that Group 2 may have spread to that region long after the initial divergence of the two groups, but before recent transfer of *E. ruminantium* to the region. We propose that this transfer was contemporary with the ancient wave of livestock domestication.

The location of clearly recombinant strains can provide some information on their recent movement, because recombination requires direct contact between strains (although these could also be transported to new locations after recombination), which in *E. ruminantium* necessarily must take place by coinfection of either host or vector in the same location. For example, the atypical presence of the Southern African sequence type 22 in Group 1 suggests recent transport of the strain from West Africa to Southern Africa. The Southern African sequence type 19 which is recombinant between Group 1 and Group 2 also suggests movement from West Africa to Southern Africa. Most of the other clear mosaic sequences that represent recombination between Group 1 and Group 2 (subgroup 2A and strain types 63 and 43) are found in West Africa or the Caribbean, suggesting that the events occurred in West Africa. As it appears likely that subgroup 2D has its origins in West Africa, the best explanation for the presence of these recombinants is the mixing of Group 1 and Group 2 strains after the arrival of Group 2 in West Africa. Indeed the Group 2-like sequences in subgroup 2A tend to be most similar to those from subgroup 2D, especially the secY allele, supporting this assertion. Strain type 69 is an exception to this with a *secY* and *lipB* allele typically found in subgroup 2E, albeit including the West African strains present in that subgroup which are presumably recent transports. As noted above, the presence of a handful of West African strain types in subgroup 2E suggests recent movement from West Africa to Southern Africa or the Indian Ocean region.

Our results are in general concordance with previous studies which also found some inconsistent phylogeny and some separation of *E. ruminantium* strains into clusters throughout Africa, the Caribbean and the Indian Ocean for a limited number of strains. Previously, Allsopp et al. (2003) and Allsopp and Allsopp (2007) studied the phylogeny of 19 *E. ruminantium* strains from West, North, East, and Southern Africa and Caribbean region. Although inconsistent phylogeny and recombination was found, they observed segregation between West Africa and East-Southern Africa, as well as the introduction of a West African-like strain (Kumm1) to Southern Africa. Another study by Nakao et al. (2011) used an MLST scheme using *gltA, groEL, lepA, lipA, lipB, secY, sodB*, and *sucA* genes, and typed 25 *E. ruminantium* strains from West, North, East and Southern Africa and the Caribbean. They found no strict association between sequence types and geographical origin by using a minimum spanning tree (MST), except for four MLST sequence types from Western Africa, however their study also evidenced recombination amongst the strains, and a neighbor net analysis produced a network with a similar structure to the one from this study (Figure 1A; Nakao et al., 2011). However, a panel of five housekeeping genes was used in our study differently from Adakal et al. (2009) and Nakao et al. (2011) that used eight genes. While our data provided less than optimal resolution, given the presence of recombination and possible slow accumulation of variation, the only optimal scheme for *E. ruminantium* is whole genome analysis.

Independently of the genes used for phylogeny and typing, a similar pattern of *E. ruminantium* genetic groups appears between previous studies and our study, with the existence of *E. ruminantium* strain clusters West Africa/Caribbean and Southern/Eastern Africa. In these previous studies, no “Worldwide strain cluster” was evidenced but none of them included more than 25 strains or covered most of the *E. ruminantium* geographic distribution compared with our current study. Specifically, Caribbean and Indian Ocean strains were well represented in our sampled set and we observed clustering of Indian Ocean and Southern Africa isolates for the first time.

Although our study includes a large number of *E. ruminantium* isolates (194), sample size varied for each country. Samples mainly from Burkina Faso (West Africa), Mozambique (Southern Africa), Madagascar (Indian Ocean), and Guadeloupe (Caribbean) collected from several localities to represent sequence types circulating in each region were chosen, with 15–48 samples per country. This might have influenced the observed genetic grouping pattern; however, it is believed that the number of samples and the sampled localities in each of the countries is sufficiently high to have a good representation of the main strains circulating and to obtain a robust *E. ruminantium* genetic population structure.

In addition, strains were mostly isolated from ticks, except in Guadeloupe (Caribbean) where they came from sick ruminants. Therefore, the *E. ruminantium* strains we studied appear to represent the natural populations, including possibly adapted virulent and non-virulent clones and strains except in Guadeloupe, where virulent strains might have been over-represented (Didelot and Maiden, 2010).

### *E. ruminantium* genetic population structure is shaped by recombination

While MLST has been a useful tool in understanding the relationship and population structure of many bacterial species (Sullivan et al., 2005), it only provides a limited view of the phylogenetic signals present in the genomes of the species studied. In species where the evolutionary history of the majority of the genome is the same, the use of several genes may provide a more robust estimation of the relationship of the strains or species studied, and reduce the error in the phylogeny when applied correctly (Yang and Rannala, 2012). In a species where many regions of the genome have differing evolutionary histories due to phenomena such as recombination, the reconstruction of phylogenies based on several genes (either individually or concatenated) may not provide a useful estimation of the relationship between the organisms examined. In the case of *E. ruminantium*, a small selection of the genome (only five genes from a total of roughly 950) exhibits multiple recombination events, suggesting that recombination between strains could be extensive in this species. Thus, it must be noted that the work presented here (as in any other MLST studies) represents only the uncovering of a partial image of the true underlying relationships between *E. ruminantium* strains in terms of their diversity and population structure. The addition of more genes to such an analysis will increase the resolution of the extent of the recombination, and will most likely further differentiate strains which appear to be clonal in more limited analyses.

Apart from the housekeeping genes used for MLST, recombination has also been described for *E. ruminantium* in other genomic regions such as *map1* (Bekker et al., 2005; Hughes and French, 2007). Bekker et al. (2005) found recombination between two *map1* paralogs in *E. ruminantium* Gardel strain while studying the expression of *map1* paralogs in one vector and several non-vector tick cell lines. Another study (Hughes and French, 2007) found evidence of recombination in *map1* alleles by identifying the locus as a statistical outlier in terms of the number of synonymous substitutions found between orthologs of this gene in two different strains. These previous studies using *map1* loci demonstrate the unsuitability of the *map1* gene as a typing marker to provide a phylogeographic structure of strains because of the high polymorphic nature of the locus. For results of studies using the *map1* gene for typing or genetic diversity, recombination events could explain part of *E. ruminantium* genetic diversity and unclear phylogeny.

No studies have yet elucidated inter-strain recombination mechanisms in the intracellular bacteria *E. ruminantium*. Regardless of the absence of plasmids, phages, insertion sequences, or genes for pilus assembly, *E. ruminantium* contain the genes necessary for DNA transfer and recombination by mechanisms that are not well understood (Collins et al., 2005; Thomas and Nielsen, 2005). Although obligate intracellular bacteria do not tend to have known mobile genetic elements, studies have shown the presence of plasmids, prophage, and transposon in the genome of five genera with multiple hosts such as *Wolbachia, Coxiella, Phytoplasma, Rickettsia, Chlamydia*, and *Chlamydophila* (Bordenstein and Reznikoff, 2005; Le et al., 2014). Additionally, Ogata et al. (2005) reported the presence of conjugative plasmids in the intracellular bacterium *R. felis*, from full genome sequencing, in the order Rickettsiales (to which *E. ruminantium* belongs). Moreover, repeated non-functional genes and mobile elements such as transposon were also reported in the intracellular alpha-proteobacteria *Orientia tsutsugamushi*, reflecting gene conversion and rearrangement. Furthermore, high levels of diversity and recombination are reported from these intracellular organism (Sonthayanon et al., 2010). Interestingly, these tandem repeat copy numbers are similar to *E. ruminantium* but the differences reflect distinct genetic diversification strategies (Cho et al., 2007).

The genome of *E. ruminantium* does code for gene transfer agent (GTA) genes that produce bacteriophage-like elements that package DNA into small virus-like particles that can be exchanged between cells and present a possible mechanism for genetic transfer between strains (Lang and Beatty, 2000, 2007). These GTAs could mediate exchange of genetic material in *E. ruminantium* in the absence of other mechanisms. More studies need to be done in *E. ruminantium* to identify the mechanism of gene transfer responsible for the recombination observed between strains.

Horizontal gene transfer (HGT) events from bacteria outside of the *Ehrlichia* genus have not been identified, which is probably due to its intracellular lifestyle where it is unlikely to come into contact with bacterial species other than coinfecting strains of *E. ruminantium*. HGT is a well-known mechanism of increasing bacterial genetic diversity that allows the recipients to gain new capabilities evolved in other species, and is often responsible for the spread of bacterial virulence and defense traits between strains or even divergent species (Ochman et al., 2000). The lack of any obvious interspecific events in *E. ruminantium* begs the question as to how it generates genetic diversity and manages to evolve strategies to continually evade host immune systems. We propose that coinfecting *E. ruminantium* strains readily swap genetic material, and that the frequent recombination between strains that we evidence here could play a large role in allowing *E. ruminantium* to continually increase its genetic diversity and avoid the host immune response.

The potential importance of recombination in the generation of genetic diversity in *E. ruminantium* cannot be overstated, because it allows the rapid mixing of variants generated by natural selection in the evolutionary arms race against host immune systems in a species that appears to be otherwise closed off from potential genetic diversity derived from interspecific horizontal transfer events due to its obligate intracellular lifestyle. Inter-strain recombination may allow this bacterium to survive and adapt under various environmental conditions in both vector and host species. The propensity for recombination between strains in this species is at the origin of its diversity which induces limited cross protection between vaccinal and field strains. The recombination between two previously distantly separated groups of the species has particular potential to dramatically increase local genetic diversity in the region where it occurs, outlining the importance of strategies to limit the spread of *E. ruminantium*, even between two regions where heartwater is already present.

Recombination is probably a major driver of genetic diversity in this obligate intracellular pathogen.

Because of recombination events seen in only the small fraction of its genome so far examined, MLST can only offer a limited view of the genetic diversity and population structure of *E. ruminantium* and caution should be taken when interpreting its genetic diversity and population structure, especially using non-network based methods.

Due to the progress of sequencing technologies in terms of cost and throughput, instead of studying a limited number of genes for phylogeny and phylogeography, it would be preferable to sequence the whole genomes of a large number of *E. ruminantium* strains with the sequencing of isolated strains in culture. Furthermore, the isolation of strains in cell culture would allow the measurement and association of phenotypic characteristics of strains with their sequence types, virulence and cross protection between strains. Identification of both recombination events in relevant loci as well as conserved genes that do not show signs of recombination may support the development of effective control strategies and the development of vaccines for *E. ruminantium*.

## Author contributions

NC, VP, LB, KH, and RA generated sequence data. JG and NC performed analysis. NC, JG, LN, DM, and NV interpreted results and wrote the manuscript. TL, NV, and LN designed the project. All authors critically reviewed and approved the final manuscript.

## Funding

This work was financially supported by CIRAD and EPIGENESIS project which received funding from the European Union's Seventh Framework Programme for research, technological development and demonstration under grant agreement No. 31598. FUNDO ABERTO DA UEM 2012-2013 and FUNDO NACIONAL DE INVESTIGAÇÃO Projecto N° 133-Inv/FNI/ 2012-2013 funded the field trips and reagents in Mozambique. French ministry of Agriculture and “Direction de l'Alimentation, de l'Agriculture et de la Forêt de Guadeloupe” financed RESPANG work. This study was partly developed under the project MALIN “Surveillance, diagnosis, control and impact of infectious diseases of humans, animals and plants in tropical islands” supported by the European Union in the framework of the European Regional Development Fund (ERDF) and the Regional Council of Guadeloupe.

### Conflict of interest statement

The authors declare that the research was conducted in the absence of any commercial or financial relationships that could be construed as a potential conflict of interest.
