# Supplementary material for: Recombination Is a Major Driving Force of Genetic Diversity in the Anaplasmataceae Ehrlichia ruminantium
Source: Front Cell Infect Microbiol. 2016 Sep 29;6:111. doi: 10.3389/fcimb.2016.00111 (PMC5040723; doi:10.3389/fcimb.2016.00111)
Supplement: Supplementary file 1 [file DataSheet1.docx]

**SUPPLEMENTARY MATERIAL**

**Figure S1** Maximum likelihood phylogeny constructed with PhyML under a GTR+G+I model of evolution with (a) and without (b) *E. chaffeensis* as an outgroup. Branch support was calculated using the aLRT method, and low support values are possibly indicative of short branches or mixed phylogenetic signals in the data, potentially introduced by recombination or other forms of homoplasy. The outgroup branch is not to scale to allow legibility of the figure.

**Figure S2** Heat map of similarity and differences of 5 concatenated housekeeping genes among 97 unique *E. ruminantium* sequences. Hypothetical groups and subgroups 1, 2A, G2B, 2C, 2D and 2D are marked by dashes. Degree of relatedness is indicated by colours from white (different) to red (similar). The name of each isolate was labelled on the right side of the graphs and corresponds to the same strains on the bottom of the graph. Dendograms representing the clusters were placed on the left side and on top of the graphs. Strains 3, 19, 42, 43, 50, 56, 63, 69, 72 and 74 are marked with a square and represent recombinants.

**Figure S3** Multiple sequence alignment of the different genotypes containing only variable positions. The subgroups clusters are separated by horizontal gaps, and the five different genes are boxed and separated by vertical gaps. Coloured residues represent non-consensus characters over the whole alignment. Blue stars indicate recombinant genotypes and red stars represent sequence types that are inferred to have less than 80% ancestry from a single population in STRUCTURE.

**Table S1** Number of *E. ruminantium* isolates/strains per geographic region and country

| **Geographic region** | **Country** | **Number of isolates** | **Total** |
| --- | --- | --- | --- |
| North and Central | Chad | 1 |  |
| Africa | Sudan | 1 | 2 |
| West Africa | Burkina Faso | **44** |  |
|  | Cameroon | 1 |  |
|  | Gambia | 1 |  |
|  | Ghana | 2 |  |
|  | Nigeria | 2 |  |
|  | São Tome and Principe | 1 |  |
|  | Senegal | 4 | 55 |
| East Africa | Kenya | 1 |  |
|  | Tanzania | 1 |  |
|  | Uganda | 1 | 3 |
| Southern Africa | Mozambique | **58** |  |
|  | South Africa | 4 |  |
|  | Zambia | 1 |  |
|  | Zimbabwe | 1 | 64 |
| Indian Ocean | Comoros | 9 |  |
|  | Madagascar | **13** |  |
|  | Mayotte | 5 |  |
|  | Reunion | 2 | 29 |
| Caribbean | Antigua | 1 |  |
|  | Guadeloupe | **40** | 41 |
|  |  |  | **194** |

**Table S2** Description of *E. ruminantium* isolates/strains based on the sequence type number, genetic group, geographic origin, country of isolation, isolate name, DNA origin, date of isolation and reference.

| **Sequence type number** | **Genetic group** | **Geographic origin** | **Country** | **Isolate/Strain**  **name** | **DNA origin** | **Date of isolation** | **Isolate**  **Reference** |
| --- | --- | --- | --- | --- | --- | --- | --- |
| 1 | G2D | East Africa | Kenya | **Kiswani** | B | 1985 | Raliniaina et al. (2010) |
| 2 | G2D | Central Africa | Chad | TCH6 | T | 2008 | Our study |
| 3 | No group | Southern Africa | South Africa | SAZeerust | CC | 1979 | Nakao et al. (2011) |
| 4 | G2C | Southern Africa | Mozambique | GAH1MH2 | T | 2012 | Our study |
| 4* | G2C |  |  | GAH4MH1 | T | 2012 | Our study |
| 5 | G2C |  |  | CIT9MH1 | T | 2012 | Our study |
| 5* | G2C |  |  | GAH7MH1 | T | 2012 | Our study |
| 5* | G2C |  |  | MWAB11FH1 | T | 2012 | Our study |
| 5* | G2C |  |  | CIT28MH1 | T | 2012 | Our study |
| 5* | G2C |  |  | Umpala | B | 1995 | Raliniaina et al. (2010) |
| 5* | G2C |  |  | MWAB11MH1 | T | 2012 | Our study |
| 5* | G2C |  |  | MAT14MH2 | T | 2012 | Our study |
| 5* | G2C |  |  | MAS13MH1 | T | 2012 | Our study |
| 6 | G2E |  |  | CHIPO29MH1 | T | 2012 | Our study |
| 6* | G2E |  |  | ZIM15MH1 | T | 2012 | Our study |
| 7 | G2E |  |  | CHIPO17MH1 | T | 2012 | Our study |
| 8 | G2E |  |  | MAS27MH2 | T | 2012 | Our study |
| 8* | G2E |  |  | ZIM31MH1 | T | 2013 | Our study |
| 9 | G2E |  |  | CHIPO12MH1 | T | 2012 | Our study |
| 10 | G2C |  |  | MAH12MH1 | T | 2012 | Our study |
| 10* | G2C |  |  | VUL29MH1 | T | 2012 | Our study |
| 10* | G2C |  |  | MWAB3MH1 | T | 2012 | Our study |
| 10* | G2C |  |  | ZIM28MH1 | T | 2012 | Our study |
| 10* | G2C |  |  | Crystal Springs | CC | 1990 | Nakao et al. (2011) |
| 11 | G2C |  |  | MAT13MH2 | T | 2012 | Our study |
| 12 | G2E |  |  | ZIM16MH2 | T | 2012 | Our study |
| 13 | G2E |  |  | VUL17MH1 | T | 2012 | Our study |
| 14 | G2E |  |  | CHIPO22MH1 | T | 2012 | Our study |
| 15 | G2E |  |  | GAH5MH2 | T | 2012 | Our study |
| 16 | G2E |  |  | CHIPO2MH1 | T | 2012 | Our study |
| 16* | G2E |  |  | CHIPA3MH1 | T | 2012 | Our study |
| 17 | G2E |  |  | ZIM2MH1 | T | 2012 | Our study |
| 17* | G2E |  |  | ZIM4MH1 | T | 2012 | Our study |
| 18 | G2E |  |  | GAH9MH1 | T | 2012 | Our study |
| 18* | G2E | Southern Africa | South Africa | SABall3 | CC | 1952 | Nakao et al. (2011) |
| 19 | No group | Southern Africa | South Africa | **Mara** | CC | 1998 | Raliniaina et al. (2010) |
| 20 | G2E | Southern Africa | Mozambique | MAH6MH1 | T | 2012 | Our study |
| 21 | G2C |  |  | MAS14MH1 | T | 2012 | Our study |
| 22 | G1 |  |  | 303-GOV1-MV8 | T | 2012 | Our study |
| 23 | G2E |  |  | 335-MAMMV13 | T | 2013 | Our study |
| 24 | G2E |  |  | 445-MAP32MH1 | T | 2013 | Our study |
| 25 | G2E |  |  | 550-NHAMV12 | T | 2014 | Our study |
| 26 | G2E |  |  | 559-NHAMV21 | T | 2014 | Our study |
| 27 | G2E |  |  | 330-MAMMV8 | T | 2013 | Our study |
| 28 | G2E |  |  | 347-MAMMV22 | T | 2013 | Our study |
| 29 | G2E |  |  | 319-MAMMV2 | T | 2013 | Our study |
| 30 | G2E |  |  | 336-MAMFV6 | T | 2013 | Our study |
| 31 | G2E |  |  | 342-MAMMV17 | T | 2013 | Our study |
| 32 | G2D |  |  | 709-DAR5MV1 | T | 2014 | Our study |
| 33 | G2E |  |  | 765-ESP2-4MV1 | T | 2014 | Our study |
| 34 | G2E |  |  | 777-DAC8MH1 | T | 2014 | Our study |
| 35 | G2E |  |  | 823-MAG7MH1 | T | 2014 | Our study |
| 36 | G2C |  |  | 431-MAP21MH1 | T | 2013 | Our study |
| 37 | G2C |  |  | 832-MAG12MH1 | T | 2014 | Our study |
| 38 | G2C |  |  | 801-MUE3FH1 | T | 2014 | Our study |
| 39 | G2D |  |  | 488-FINMV13 | T | 2014 | Our study |
| 40 | G2D |  |  | 595-MANINMV19 | T | 2014 | Our study |
| 41 | G2D |  |  | 385-MUX3MV1 | T | 2013 | Our study |
| 42 | G2D |  |  | 394-MUX9MV1 | T | 2013 | Our study |
| 43 | No group | West Africa | Burkina Faso | Sara401 | T | 2002 | Raliniaina et al. (2010) |
| 44 | G2D |  |  | Lamba194 | B | 2003 | Raliniaina et al. (2010) |
| 44* | G2D |  |  | **Banankeledaga** | CC | 1998 | Raliniaina et al. (2010) |
| 44* | G2D |  |  | BF629 | T | 2009 | Adakal et al. (2010) |
| 44* | G2D |  |  | BF630 | T | 2009 | Adakal et al. (2010) |
| 44* | G2D |  |  | BF635 | T | 2009 | Adakal et al. (2010) |
| 45 | G2D |  |  | Banan455 | B | 2003 | Raliniaina et al. (2010) |
| 46 | G1 | West Africa | Senegal | M310 | T | 2002 | Our study |
| 47 | G2D | West Africa | Burkina Faso | Banan033F1 | T | 2002 | Raliniaina et al. (2010) |
| 48 | G1 | West Africa | Ghana | Sankat430 | CC | 1996 | Nakao et al. (2011) |
| 48* | G1 | West Africa | Burkina Faso | BF1210 | T | 2007 | Adakal et al. (2010) |
| 48* | G1 |  |  | BF1795 | T | 2007 | Adakal et al. (2010) |
| 48* | G1 |  |  | BF1796 | T | 2007 | Adakal et al. (2010) |
| 48* | G1 |  |  | BF1798 | T | 2007 | Adakal et al. (2010) |
| 48* | G1 |  |  | BF19 | T | 2007 | Adakal et al. (2010) |
| 49 | G1 | West Africa | Gambia | Kerr Seringe | CC | 2001 | Nakao et al. (2011) |
| 49* | G1 | West Africa | Senegal | M10T | T | 2002 | Raliniaina et al. (2010) |
| 50 | G2A |  |  | M16T | T | 2002 | Raliniaina et al. (2010) |
| 51 | G2B | West Africa | Burkina Faso | BF623 | T | 2007 | Adakal et al. (2010) |
| 52 | G1 | West Africa | Nigeria | SK43M1 | T | 2010 | Our study |
| 53 | G1 | West Africa | Burkina Faso | BF331 | T | 2007 | Adakal et al. (2010) |
| 54 | G2E | West Africa | São Tome and Principe | São Tome | CC | 1981 | Nakao et al. (2011) |
| 55 | G2D | West Africa | Burkina Faso | BF1042 | T | 2007 | Adakal et al. (2010) |
| 56 | G2A |  |  | BF1062 | T | 2007 | Adakal et al. (2010) |
| 57 | G2E |  |  | BF1232 | T | 2007 | Adakal et al. (2010) |
| 58 | G2B |  |  | BF1267 | T | 2007 | Adakal et al. (2010) |
| 59 | G1 |  |  | BF1799 | T | 2007 | Adakal et al. (2010) |
| 60 | G1 |  |  | BF1905 | T | 2007 | Adakal et al. (2010) |
| 61 | G1 |  |  | BF1948 | T | 2007 | Adakal et al. (2010) |
| 62 | G2D |  |  | BF1951 | T | 2007 | Adakal et al. (2010) |
| 63 | No group |  |  | BF2185 | T | 2007 | Adakal et al. (2010) |
| 64 | G2B |  |  | BF631 | T | 2007 | Adakal et al. (2010) |
| 65 | G2E |  |  | BF668 | T | 2007 | Adakal et al. (2010) |
| 66 | G2E |  |  | BF708 | T | 2007 | Adakal et al. (2010) |
| 67 | G2D | Caribbean | Antigua | GeorgesM3 | T | 2005 | Raliniaina et al. (2010) |
| 68 | G2D | Caribbean | Guadeloupe | 34-0205CM01 | B | 2011 | Our study |
| 68* | G2D |  |  | **Gardel** | CC | 1962 | Raliniaina et al. (2010) |
| 68* | G2D |  |  | 27-2103JMR03 | B | 2011 | Our study |
| 69 | G2A |  |  | 11-1711BP02 | B | 2010 | Our study |
| 70 | G2D |  |  | 25-2103JMR01 | B | 2011 | Our study |
| 70* | G2D |  |  | 49-250112VL01 | B | 2012 | Our study |
| 70* | G2D |  |  | 38-0507AS01 | B | 2011 | Our study |
| 70* | G2D |  |  | 46-061211JCA01 | B | 2011 | Our study |
| 70* | G2D |  |  | SUI22M1B1 | B | 2008 | Our study |
| 70* | G2D |  |  | n6631 | B | 2009 | Our study |
| 71 | G2D |  |  | 35-0805JMR01 | B | 2011 | Our study |
| 71* | G2D |  |  | n5697 | B | 2005 | Our study |
| 71* | G2D |  |  | n5097 | B | 2002 | Our study |
| 72 | G2A |  |  | 13-2112JCA01 | B | 2011 | Our study |
| 72* | G2A |  |  | SUI24JM | B | 2008 | Our study |
| 72* | G2A |  |  | 14-2112JCA02 | B | 2011 | Our study |
| 72* | G2A |  |  | 6-2709EH03 | B | 2010 | Our study |
| 72* | G2A |  |  | 48-291111FB01 | B | 2011 | Our study |
| 72* | G2A |  |  | n971128610M2 | B | 2005 | Our study |
| 72* | G2A |  |  | 15-2112JCA03 | B | 2011 | Our study |
| 73 | G2E |  |  | 33-2704AS01 | B | 2011 | Our study |
| 73* | G2E |  |  | 42-1509JE01 | B | 2011 | Our study |
| 73* | G2E |  |  | 39-2008FB01 | B | 2011 | Our study |
| 73* | G2E |  |  | 32-1104FB01 | B | 2011 | Our study |
| 73* | G2E |  |  | 40-2408FB02 | B | 2011 | Our study |
| 74 | G2A |  |  | 19-0202BP01 | B | 2011 | Our study |
| 75 | G2B |  |  | 44-2110JE01 | B | 2011 | Our study |
| 75* | G2B |  |  | 26-2103JMR02 | B | 2011 | Our study |
| 75* | G2B |  |  | 43-2610VL01 | B | 2011 | Our study |
| 76 | G2D |  |  | 36-1405BP01 | B | 2011 | Our study |
| 76* | G2D |  |  | 30-1304MC01 | B | 2011 | Our study |
| 77 | G2D |  |  | 45-161111MM01 | B | 2011 | Our study |
| 77* | G2D |  |  | n6001 | B | 2000 | Our study |
| 77* | G2D |  |  | 29-2903JMR01 | B | 2011 | Our study |
| 78 | G2D |  |  | n6653/1-2313 | B | 2002 | Our study |
| 79 | G2D | Indian Ocean | Comoros | AY0024 | T | 2010 | Our study |
| 80 | G2E | Indian Ocean | Reunion | APLSM1 | T | 2010 | Our study |
| 81 | G2D | Indian Ocean | Madagascar | Madaman1 | T | 2008 | Our study |
| 82 | G2E | Indian Ocean | Comoros | n1690 | T | 2007 | Our study |
| 83 | G2D | Indian Ocean | Madagascar | n13BM1 | T | 2010 | Our study |
| 84 | G2E | Indian Ocean | Mayotte | n164B2458 | T | 2010 | Our study |
| 85 | G2E | Indian Ocean | Madagascar | n8EM3 | T | 2010 | Our study |
| 86 | G2E | Indian Ocean | Comoros | AY0015 | T | 2010 | Our study |
| 87 | G2D |  |  | AY0041 | T | 2010 | Our study |
| 88 | G2E | Indian Ocean | Mayotte | TiquesM3 | T | 2010 | Our study |
| 88* | G2E | Indian Ocean | Madagascar | n8DF3 | T | 2010 | Our study |
| 88* | G2E | Indian Ocean | Mayotte | n206B | T | 2010 | Our study |
| 88* | G2E |  |  | YTBARA8M1 | T | 2009 | Our study |
| 89 | G2E | Indian Ocean | Madagascar | n2CM4 | T | 2009 | Our study |
| 90 | G2E |  |  | n14AF1 | T | 2010 | Our study |
| 91 | G2E | Indian Ocean | Comoros | AY0091 | T | 2010 | Our study |
| 91* | G2E |  |  | AY0087 | T | 2010 | Our study |
| 92 | G2E | Indian Ocean | Madagascar | KJSF | T | 2001 | Our study |
| 92* | G2E |  |  | Madaman3 | T | 2008 | Our study |
| 92* | G2E |  |  | n14CM1 | T | 2010 | Our study |
| 92* | G2E |  |  | Madaman13 | T | 2008 | Our study |
| 92* | G2E | Indian Ocean | Comoros | n3700 | T | 2007 | Our study |
| 92* | G2E |  |  | n3683 | T | 2007 | Our study |
| 92* | G2E | Indian Ocean | Reunion | BDLSM3 | T | 2010 | Our study |
| 92* | G2E | Indian Ocean | Madagascar | Madaman4 | T | 2008 | Our study |
| 92* | G2E | Indian Ocean | Comoros | n3655 | T | 2007 | Our study |
| 92* | G2E | Indian Ocean | Madagascar | RZF | T | 2001 | Raliniaina et al. (2010) |
| 93 | G2E | Southern Africa | Mozambique | CHIPO26MH1 | T | 2012 | Our study |
| 93* | G2E |  |  | CHIPA2MH1 | T | 2012 | Our study |
| 93* | G2E | West Africa | Cameroon | Cameroun | CC | 1994 | Raliniaina et al. (2010) |
| 93* | G2E | Southern Africa | Mozambique | MAS1MH1 | T | 2012 | Our study |
| 93* | G2E | Southern Africa | South Africa | **Welgevonden** | CC | 1985 | Raliniaina et al. (2010) |
| 93* | G2E | Southern Africa | Mozambique | CHIPO24MH2 | T | 2012 | Our study |
| 93* | G2E |  |  | ZIM1MH1 | T | 2012 | Our study |
| 94 | G2E | Southern Africa | Zambia | **Lutale** | CC | 1988 | Raliniaina et al. (2010) |
| 94* | G2E | North Africa | Sudan | **Umbanein** | CC | 1981 | Raliniaina et al. (2010) |
| 94* | G2E | Indian Ocean | Madagascar | Madamora3 | T | 2008 | Our study |
| 94* | G2E | East Africa | Uganda | KBL4M | T | 1999 | Our study |
| 94* | G2E | Indian Ocean | Mayotte | YTAVI001 | T | 2009 | Our study |
| 95 | G2D | West Africa | Nigeria | NigeriaIfe | B | 1983 | Nakao et al. (2011) |
| 95* | G2D | Caribbean | Guadeloupe | 4-2007AS02 | T | 2010 | Our study |
| 95* | G2D | West Africa | Burkina Faso | BF2 | T | 2007 | Adakal et al. (2010) |
| 95* | G2D | Caribbean | Guadeloupe | 37-0806FB01 | T | 2011 | Our study |
| 95* | G2D |  |  | 21-2702VL01 | T | 2011 | Our study |
| 95* | G2D |  |  | 41-0309FB01 | T | 2011 | Our study |
| 96 | G1 | East Africa | Tanzania | AB014TAN | T | 2010 | Our study |
| 96* | G1 | West Africa | Ghana | **Pokoase** | CC | 1996 | Raliniaina et al. (2010) |
| 97 | G1 | West Africa | Burkina Faso | lamba479 | T | 2001 | Raliniaina et al. (2010) |
| 97* | G1 | Caribbean | Guadeloupe | 17-2701GM01 | T | 2011 | Our study |
| 97* | G1 | West Africa | Burkina Faso | bankouma421 | T | 2001 | Raliniaina et al. (2010) |
| 97* | G1 | West Africa | Senegal | **Senegal** | CC | 1994 | Raliniaina et al. (2010) |
| 97* | G1 | West Africa | Burkina Faso | Sara292 | T | 2001 | Raliniaina et al. (2010) |
| 97* | G1 |  |  | Lamba107 | T | 2002 | Raliniaina et al. (2010) |
| 97* | G1 |  |  | Bekuy255 | CC | 2001 | Raliniaina et al. (2010) |
| 97* | G1 |  |  | BF395 | T | 2007 | Adakal et al. (2010) |
| 97* | G1 |  |  | BF1114 | T | 2007 | Adakal et al. (2010) |
| 97* | G1 |  |  | BF1946 | T | 2007 | Adakal et al. (2010) |
| 97* | G1 |  |  | BF2165 | T | 2007 | Adakal et al. (2010) |
| 97* | G1 |  |  | BF461 | T | 2007 | Adakal et al. (2010) |
| 97* | G1 |  |  | BF463 | T | 2007 | Adakal et al. (2010) |
| 97* | G1 |  |  | BF466 | T | 2007 | Adakal et al. (2010) |
| 97* | G1 |  |  | BF469 | T | 2007 | Adakal et al. (2010) |
| 97* | G1 |  |  | BF474 | T | 2008 | Adakal et al. (2010) |
| 97* | G1 |  |  | BF476 | T | 2007 | Adakal et al. (2010) |
| 97* | G1 |  |  | BF810 | T | 2007 | Adakal et al. (2010) |

*Identical DNA sequence (clone). Reference strains are highlighted in bold. CC: Cell culture; T: Tick, B: Blood.

**Table S3** Number of *E. ruminantium* isolates per genetic group and country

| **Group** | **Country** | **Number of samples** | **Total** |
| --- | --- | --- | --- |
| 1 | Burkina Faso | **25** |  |
|  | Gambia | 1 |  |
|  | Ghana | 2 |  |
|  | Guadeloupe | 1 |  |
|  | Mozambique | 1 |  |
|  | Nigeria | 1 |  |
|  | Senegal | 3 |  |
|  | Tanzania | 1 | 35 |
| 2A | Burkina Faso | 1 |  |
|  | Guadeloupe | **9** |  |
|  | Senegal | 1 | 11 |
| 2B | Burkina Faso | 3 |  |
|  | Guadeloupe | 3 | 6 |
| 2C | Mozambique | **19** |  |
|  | Zimbabwe | 1 | 20 |
| 2D | Antigua | 1 |  |
|  | Burkina Faso | 10 |  |
|  | Chad | 1 |  |
|  | Comoros | 2 |  |
|  | Guadeloupe | **22** |  |
|  | Kenya | 1 |  |
|  | Madagascar | 2 |  |
|  | Mozambique | 5 |  |
|  | Nigeria | 1 |  |
|  | South Africa | 1 | 46 |
| 2E | Burkina Faso | 3 |  |
|  | Cameroon | 1 |  |
|  | Comoros | 7 |  |
|  | Guadeloupe | 5 |  |
|  | Madagascar | 11 |  |
|  | Mayotte | 5 |  |
|  | Mozambique | **33** |  |
|  | Reunion | 2 |  |
|  | São Tome e Principe | 1 |  |
|  | South Africa | 2 |  |
|  | Sudan | 1 |  |
|  | Uganda | 1 |  |
|  | Zambia | 1 | 73 |
| No group | Burkina Faso | **2** |  |
|  | South Africa | 1 | 3 |
| **Total** |  |  | **194** |

**Table S4 GeneBank accession number corresponding to each gene sequence (order *sucA*-*sodB*-*lipA*-*secY*-*lipB*) for 67 *E. ruminantium* sequence type**

| **Sequence type number** | **Accession number** | | | | |
| --- | --- | --- | --- | --- | --- |
|  | **sucA** | **sodB** | **lipA** | **secY** | **lipB** |
| 1 | KX821405 | KX821339 | KX889850 | KX821470 | KX821537 |
| 2 | KX821406 | KX821340 | KX889851 | KX821471 | KX821538 |
| 3 | KX821407 | KX821341 | KX889852 | KX821472 | KX821539 |
| 4 | KX821408 | KX821342 | KX889853 | KX821473 | KX821540 |
| 5 | KX821409 | KX821343 | KX889854 | KX821474 | KX821541 |
| 6 | KX821410 | KX821344 | KX889855 | KX821475 | KX821542 |
| 7 | KX821411 | KX821345 | KX889856 | KX821476 | KX821543 |
| 8 | KX821412 | KX821346 | KX889857 | KX821477 | KX821544 |
| 9 | KX821413 | KX821347 | KX889858 | KX821478 | KX821545 |
| 10 | KX821414 | KX821348 | KX889859 | KX821479 | KX821546 |
| 11 | KX821415 | KX821349 | KX889860 | KX821480 | KX821547 |
| 12 | KX821416 | KX821350 | KX889861 | KX821481 | KX821548 |
| 13 | KX821417 | KX821351 | KX889862 | KX821482 | KX821549 |
| 14 | KX821418 | KX821352 | KX889863 | KX821483 | KX821550 |
| 15 | KX821419 | KX821353 | KX889864 | KX821484 | KX821551 |
| 16 | KX821420 | KX821354 | KX889865 | KX821485 | KX821552 |
| 17 | KX821421 | KX821355 | KX889866 | KX821486 | KX821553 |
| 18 | KX821422 | KX821356 | KX889867 | KX821487 | KX821554 |
| 19 | KX821423 | KX821357 | KX889868 | KX821488 | KX821555 |
| 20 | KX821424 | KX821358 | KX889869 | KX821489 | KX821556 |
| 21 | KX821425 | KX821359 | KX889870 | KX821490 | KX821557 |
| 22 | KX821426 | KX821360 | KX889871 | KX821491 | KX821558 |
| 23 | KX821427 | KX821361 | KX889872 | KX821492 | KX821559 |
| 24 | KX821428 | KX821362 | KX889873 | KX821493 | KX821560 |
| 25 | KX821429 | KX821363 | KX889874 | KX821494 | KX821561 |
| 26 | KX821430 | KX821364 | KX889875 | KX821495 | KX821562 |
| 27 | KX821431 | KX821365 | KX889876 | KX821496 | KX821563 |
| 28 | KX821432 | KX821366 | KX889877 | KX821497 | KX821564 |
| 29 | KX821433 | KX821367 | KX889878 | KX821498 | KX821565 |
| 30 | KX821434 | KX821368 | KX889879 | KX821499 | KX821566 |
| 31 | KX821435 | KX821369 | KX889880 | KX821500 | KX821567 |
| 32 | KX821436 | KX821370 | KX889881 | KX821501 | KX821568 |
| 33 | KX821437 | KX821371 | KX889882 | KX821502 | KX821569 |
| 34 | KX821438 | KX821372 | KX889883 | KX821503 | KX821570 |
| 35 | KX821439 | KX821373 | KX889884 | KX821504 | KX821571 |
| 36 | KX821440 | KX821374 | KX889885 | KX821505 | KX821572 |
| 37 | KX821441 | KX821375 | KX889886 | KX821506 | KX821573 |
| 38 | KX821442 | KX821376 | KX889887 | KX821507 | KX821574 |
| 39 | KX821443 | KX821377 | KX889888 | KX821508 | KX821575 |
| 40 | KX821444 | KX821378 | KX889889 | KX821509 | KX821576 |
| 41 | KX821445 | KX821379 | KX889890 | KX821510 | KX821577 |
| 42 | KX821446 | KX821380 | KX889891 | KX821511 | KX821578 |
| 43 | KX821447 | KX821381 | KX889892 | KX821512 | KX821579 |
| 44 | KX821448 | KX821382 | KX889893 | KX821513 | KX821580 |
| 45 | KX821449 | KX821383 | KX889894 | KX821514 | KX821581 |
| 46 | KX821450 | KX821384 | KX889895 | KX821515 | KX821582 |
| 47 | KX821451 | KX821385 | KX889896 | KX821516 | KX821583 |
| 48 | KX821452 | KX821386 | KX889897 | KX821517 | KX821584 |
| 49 | KX821453 | KX821387 | KX889898 | KX821518 | KX821585 |
| 50 | KX821454 | KX821388 | KX889899 | KX821519 | KX821586 |
| 51 | KX821455 | KX821389 | KX889900 | KX821520 | KX821587 |
| 52 | KX821456 | KX821390 | KX889901 | KX821521 | KX821588 |
| 53 | KX821457 | KX821391 | KX889902 | KX821522 | KX821589 |
| 54 | KX821458 | KX821392 | KX889903 | KX821523 | KX821590 |
| 55 | KX821459 | KX821393 | KX889904 | KX821524 | KX821591 |
| 56 | KX821460 | KX821394 | KX889905 | KX821525 | KX821592 |
| 57 | KX821461 | KX821395 | KX889906 | KX821526 | KX821593 |
| 58 | KX821462 | KX821396 | KX889907 | KX821527 | KX821594 |
| 59 | KX821463 | KX821397 | KX889908 | KX821528 | KX821595 |
| 60 | KX821464 | KX821398 | KX889909 | KX821529 | KX821596 |
| 61 | KX821465 | KX821399 | KX889910 | KX821530 | KX821597 |
| 62 | KX821466 | KX821400 | KX889911 | KX821531 | KX821598 |
| 63 | KX821467 | KX821401 | KX889912 | KX821532 | KX821599 |
| 64 | KX821468 | KX821402 | KX889913 | KX821533 | KX821600 |
| 65 | KX821469 | KX821403 | KX889914 | KX821534 | KX821601 |
| 66 | x^a^ | KX821404 | KX889915 | KX821535 | KX821602 |
| 67 | x^a^ | x^a^ | KX889916 | KX821536 | KX821603 |

^a^: Not possible to have accession number for this DNA sequences
