# Supplementary figures and images for: Recombination Is a Major Driving Force of Genetic Diversity in the Anaplasmataceae Ehrlichia ruminantium
Source: Front Cell Infect Microbiol. 2016 Sep 29;6:111. doi: 10.3389/fcimb.2016.00111 (PMC5040723; doi:10.3389/fcimb.2016.00111)

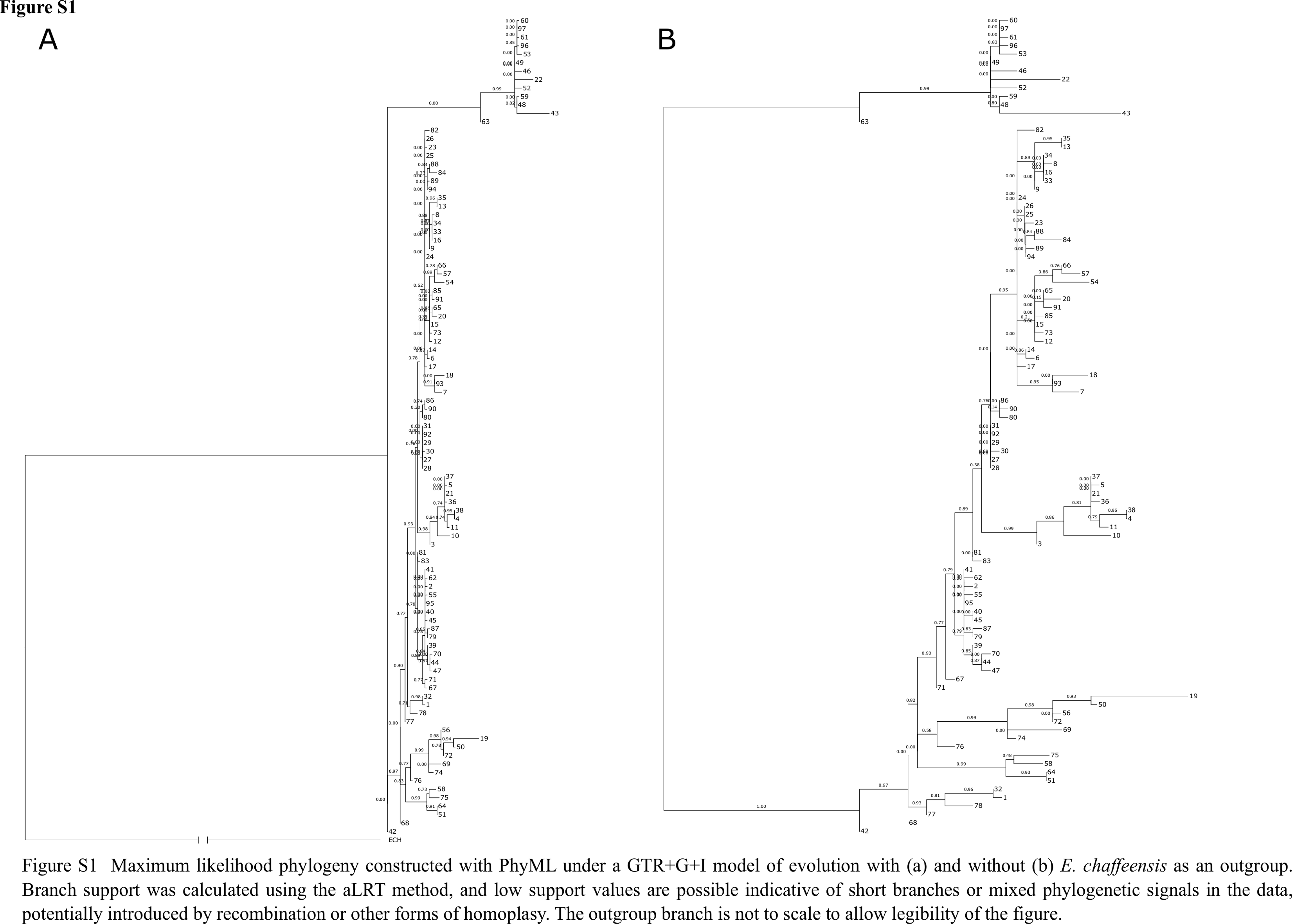

Supplement: Supplementary file 2 [file Image1.tif]

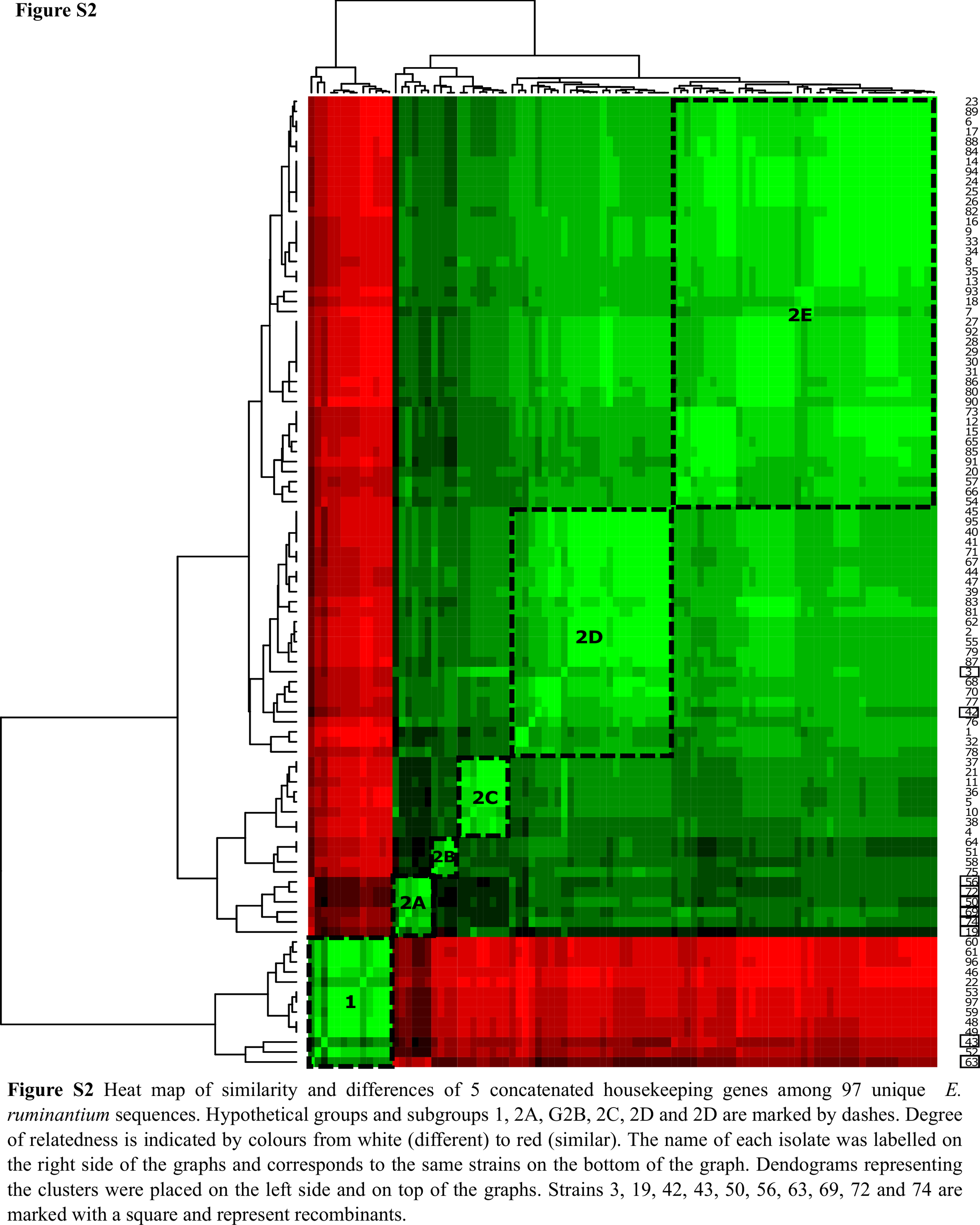

Supplement: Supplementary file 3 [file Image2.tif]

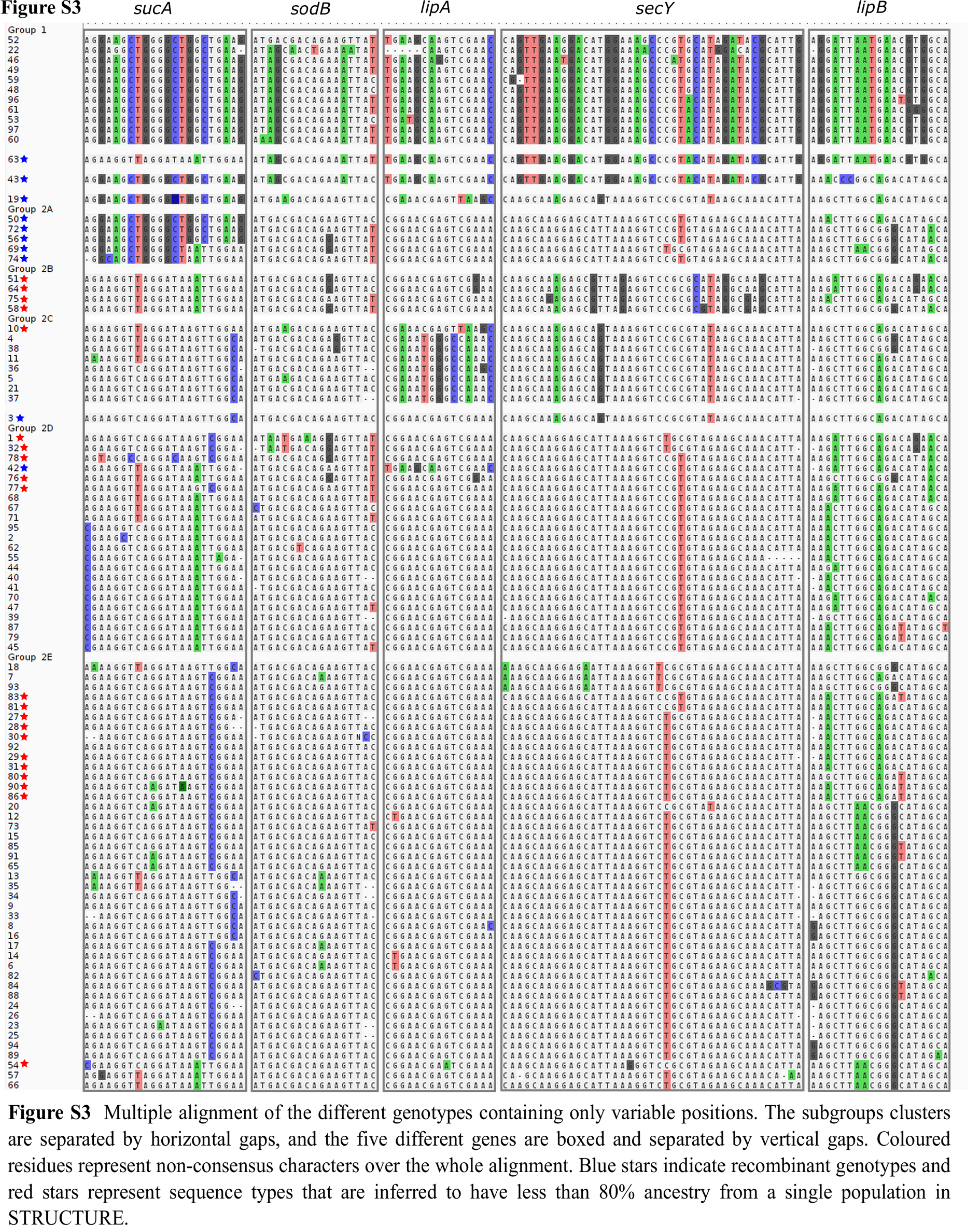

Supplement: Supplementary file 4 [file Image3.tif]
